# Supplementary material for: Atmospheric impregnation behavior of calcium phosphate materials for antibiotic therapy in neurotrauma surgery
Source: PLoS One. 2020 Mar 17;15(3):e0230533. doi: 10.1371/journal.pone.0230533 (PMC7077826; doi:10.1371/journal.pone.0230533)
Supplement: S1 File — (PDF) [file pone.0230533.s001.pdf]

Supporting information for raw data

Atmospheric impregnation behavior of calcium phosphate materials for antibiotic therapy in neurotrauma surgery

Akihito Kato*

Department of Emergency, Disaster, and Critical Care Medicine, Showa University of Medicine, Tokyo, Japan

Impregnation under decompression

CP-A	W_b (g)	W_a (g)	$W_a - W_b$ (g)	W_r (g/g)	Average
1	0.4618	1.2592	0.7974	1.7267	
2	0.4575	1.2418	0.7843	1.7143	1.6442
3	0.5321	1.3258	0.7937	1.4916	

CP-B	W_b (g)	W_a (g)	$W_a - W_b$ (g)	W_r (g/g)	Average
1	0.7777	1.4276	0.6499	0.8357	
2	0.7720	1.4167	0.6447	0.8351	0.8317
3	0.7948	1.4500	0.6552	0.8244	

CP-C	W_b (g)	W_a (g)	$W_a - W_b$ (g)	W_r (g/g)	Average
1	1.5280	2.3679	0.8399	0.5497	
2	1.6093	2.4089	0.7996	0.4969	0.5393
3	1.4675	2.3058	0.8383	0.5712	

CP-D	W_b (g)	W_a (g)	$W_a - W_b$ (g)	W_r (g/g)	Average
1	1.3747	1.8345	0.4598	0.3345	
2	1.3988	1.8751	0.4763	0.3405	0.3402
3	1.3657	1.8378	0.4721	0.3457	

CP-E	W_b (g)	W_a (g)	$W_a - W_b$ (g)	W_r (g/g)	Average
1	1.4773	1.9103	0.4330	0.2931	
2	1.4581	1.8940	0.4359	0.2990	0.3027
3	1.4772	1.9439	0.4667	0.3159	

Atmospheric impregnation

CP-A	W_b (g)	W_a (g)	W_a - W_b(g)	W_s (g/g)	R_s (%)
15 min-1	0.4803	1.2286	0.7483	1.5580	94.8
15 min-2	0.4704	1.1676	0.6972	1.4821	90.1
15 min-3	0.4531	1.2289	0.7758	1.7122	104.1
30 min-1	0.4823	1.2464	0.7641	1.5843	96.4
30 min-2	0.4474	1.1778	0.7304	1.6325	99.3
30 min-3	0.4511	1.1929	0.7418	1.6444	100.0
60 min-1	0.4774	1.1552	0.6778	1.4198	86.3
60 min-2	0.4658	1.2324	0.7666	1.6458	100.1
60 min-3	0.4612	1.1746	0.7134	1.5468	94.1
120 min-1	0.4617	1.1047	0.6430	1.3927	84.7
120 min-2	0.4562	1.1443	0.6881	1.5083	91.7
120 min-3	0.5317	1.2165	0.6848	1.2879	78.3

CP-B	W_b (g)	W_a (g)	W_a - W_b(g)	W_s (g/g)	R_s (%)
15 min-1	0.7705	1.3445	0.5740	0.7450	89.6
15 min-2	0.7242	1.3069	0.5827	0.8046	96.7
15 min-3	0.7764	1.3430	0.5666	0.7298	87.7
30 min-1	0.7915	1.3597	0.5682	0.7179	86.3
30 min-2	0.8040	1.3994	0.5954	0.7405	89.0
30 min-3	0.7392	1.3217	0.5825	0.7880	94.7
60 min-1	0.7894	1.3556	0.5662	0.7173	86.2
60 min-2	0.7493	1.3314	0.5821	0.7769	93.4
60 min-3	0.7562	1.3185	0.5623	0.7436	89.4
120 min-1	0.7767	1.3144	0.5377	0.6923	83.2
120 min-2	0.7704	1.3281	0.5577	0.7239	87.0
120 min-3	0.7938	1.3429	0.5491	0.6917	83.2

CP-C	W_b (g)	W_a (g)	W_a - W_b(g)	W_s (g/g)	R_s (%)
15 min-1	1.5045	2.2030	0.6985	0.4643	86.1
15 min-2	1.5057	2.2075	0.7018	0.4661	86.4
15 min-3	1.5099	2.1987	0.6888	0.4562	84.6
30 min-1	1.4529	2.1958	0.7429	0.5113	94.8
30 min-2	1.4624	2.1916	0.7292	0.4986	92.5
30 min-3	1.5131	2.2093	0.6962	0.4601	85.3
60 min-1	1.5092	2.2388	0.7296	0.4834	89.6
60 min-2	1.5220	2.2358	0.7138	0.4690	87.0
60 min-3	1.5963	2.2982	0.7019	0.4397	81.5
120 min-1	1.5254	2.2147	0.6893	0.4519	83.8
120 min-2	1.6071	2.2846	0.6775	0.4216	78.2
120 min-3	1.4660	2.1777	0.7117	0.4855	90.0

CP-D	W_b (g)	W_a (g)	W_a - W_b(g)	W_s (g/g)	R_s (%)
15 min-1	1.3790	1.7203	0.3413	0.2475	72.7
15 min-2	1.3632	1.6960	0.3328	0.2441	71.8
15 min-3	1.3527	1.6816	0.3289	0.2431	71.5
30 min-1	1.3808	1.7272	0.3464	0.2509	73.7
30 min-2	1.3603	1.7028	0.3425	0.2518	74.0
30 min-3	1.3550	1.7170	0.3620	0.2672	78.5
60 min-1	1.3691	1.7040	0.3349	0.2446	71.9
60 min-2	1.3818	1.7081	0.3263	0.2361	69.4
60 min-3	1.3947	1.7030	0.3083	0.2211	65.0
120 min-1	1.3739	1.6836	0.3097	0.2254	66.3
120 min-2	1.3977	1.7425	0.3448	0.2467	72.5
120 min-3	1.3647	1.7310	0.3663	0.2684	78.9

CP-E	W_b（g）	W_a（g）	W_a - W_b(g)	W_s（g/g）	R_s（%）
15 min-1	1.4933	1.7071	0.2138	0.1432	47.3
15 min-2	1.5663	1.7831	0.2168	0.1384	45.7
15 min-3	1.5438	1.7779	0.2341	0.1516	50.1
30 min-1	1.4907	1.7248	0.2341	0.1570	51.9
30 min-2	1.5689	1.7766	0.2077	0.1324	43.7
30 min-3	1.5712	1.7917	0.2205	0.1403	46.4
60 min-1	1.5339	1.7904	0.2565	0.1672	55.2
60 min-2	1.5495	1.7787	0.2292	0.1479	48.9
60 min-3	1.5146	1.7406	0.2260	0.1492	49.3
120 min-1	1.4771	1.6541	0.1770	0.1198	39.6
120 min-2	1.4581	1.6292	0.1711	0.1173	38.8
120 min-3	1.4769	1.7139	0.2370	0.1605	53.0

Experimental impregnation ratio (reduced pressure)

CP-A	W_b (g)	W_a (g)	W_a - W_b (g)	R_e (%)
1	0.4618	1.2592	0.7974	79.7
2	0.4575	1.2418	0.7843	78.4
3	0.5321	1.3258	0.7937	79.4

CP-B	W_b (g)	W_a (g)	W_a - W_b (g)	R_e (%)
1	0.7777	1.4276	0.6499	65.0
2	0.7720	1.4167	0.6447	64.5
3	0.7948	1.4500	0.6552	65.5

CP-C	W_b (g)	W_a (g)	W_a - W_b (g)	R_e (%)
1	1.5280	2.3679	0.8399	56.0
2	1.6093	2.4089	0.7996	53.3
3	1.4675	2.3058	0.8383	55.9

CP-D	W_b (g)	W_a (g)	W_a - W_b (g)	R_e (%)
1	1.3747	1.8345	0.4598	46.0
2	1.3988	1.8751	0.4763	47.6
3	1.3657	1.8378	0.4721	47.2

CP-E	W_b (g)	W_a (g)	W_a - W_b (g)	R_e (%)
1	1.4773	1.9103	0.4330	43.3
2	1.4581	1.8940	0.4359	43.6
3	1.4772	1.9439	0.4667	46.7

Experimental impregnation ratio (atmospheric pressure)

CP-A	W_b (g)	W_a (g)	W_a - W_b(g)	R_e (%)
15 min-1	0.4803	1.2286	0.7483	74.8
15 min-2	0.4704	1.1676	0.6972	69.7
15 min-3	0.4531	1.2289	0.7758	77.6

CP-B	W_b (g)	W_a (g)	W_a - W_b(g)	R_e (%)
15 min-1	0.7705	1.3445	0.5740	57.4
15 min-2	0.7242	1.3069	0.5827	58.3
15 min-3	0.7764	1.3430	0.5666	56.7

CP-C	W_b (g)	W_a (g)	W_a - W_b(g)	R_e (%)
15 min-1	1.5045	2.2030	0.6985	46.6
15 min-2	1.5057	2.2075	0.7018	46.8
15 min-3	1.5099	2.1987	0.6888	45.9

CP-D	W_b (g)	W_a (g)	W_a - W_b(g)	R_e (%)
15 min-1	1.3790	1.7203	0.3413	34.1
15 min-2	1.3632	1.6960	0.3328	33.3
15 min-3	1.3527	1.6816	0.3289	32.9

CP-E	W_b (g)	W_a (g)	W_a - W_b(g)	R_e (%)
15 min-1	1.4933	1.7071	0.2138	21.4
15 min-2	1.5663	1.7831	0.2168	21.7
15 min-3	1.5438	1.7779	0.2341	23.4

Theoretical impregnation ratio

CP-A	W（g）	vd（g）	R_t（%）
1	0.4617	3.38	86.3
2	0.4562	3.38	86.5
3	0.5317	3.40	84.4

CP-B	W（g）	vd（g）	R_t（%）
1	0.7767	3.19	75.7
2	0.7704	3.22	76.1
3	0.7938	3.24	75.5

CP-C	W（g）	vd（g）	R_t（%）
1	1.5254	4.75	67.9
2	1.6071	4.77	66.3
3	1.4660	4.76	69.2

CP-D	W（g）	vd（g）	R_t（%）
1	1.3739	3.23	57.4
2	1.3977	3.26	57.1
3	1.3647	3.21	57.6

CP-E	W（g）	vd（g）	R_t（%）
1	1.4771	3.18	53.6
2	1.4581	3.18	54.2
3	1.4769	3.19	53.7
